# Optimization of the preoperative requirements of blood units for the surgical treatment of extra-abdominal soft tissue sarcoma: the TRANSAR score

**DOI:** 10.1186/s12957-022-02839-0

**Published:** 2022-12-04

**Authors:** Farhad Farzaliyev, Hans-Ulrich Steinau, Atajan Halmyradov, Eugen Malamutmann, Arie Sleutel, Claudius Illg, Lars Erik Podleska

**Affiliations:** 1 Department of Hand-, Plastic, Reconstructive and Burn Surgery, BG Klinik, Eberhard Karls University Tuebingen, Schnarrenbergstrasse 95, 72076 Tuebingen, Germany; 2 Department of Tumor Orthopedics and Sarcoma Surgery, University Hospital Essen, Essen, Germany; 3 Department of Anesthesiology and Intensive Care Medicine, Evangelical Hospital Duisburg, Duisburg, Germany; 4 Department of General, Visceral and Transplantation Surgery, University Hospital of Essen, Science University Duisburg-Essen, Essen, Germany

**Keywords:** Blood transfusion, Soft tissue sarcoma, TRANSAR-Score, Risk prediction score model

## Abstract

**Background and objectives:**

Excessive preoperative blood orders frequently occur during the preoperative planning of resections of sarcomas. We aimed to develop a prediction score model that would be able to identify a patient cohort in which the cross-matching could be safely evaded.

**Patients and methods:**

We retrospectively analyzed data of 309 consecutive patients with extra-abdominal soft tissue sarcomas treated between September 2012 and December 2014. Scorecard scores for variables were calculated and summarized to a total score that can be used for risk stratification. The score was used in a logistic regression model. Results of the optimized model were described as a receiver operating characteristic curve.

**Results:**

Preoperative units of red blood cells were requested for 206 (66.7%) patients, of which only 31 (10%) received them. Five parameters were identified with high predictive power. In the visualized barplot, there was an increased risk of blood transfusion with a higher score of TRANSAR.

**Conclusion:**

A TRANSAR score is a new tool that can predict the probability of transfusion for patients with sarcoma. This may reduce the number of preoperative cross-matching and blood product ordering and associated costs without compromising patient care.

**Supplementary Information:**

The online version contains supplementary material available at 10.1186/s12957-022-02839-0.

## Introduction

Despite improvements in the perioperative strategies to minimize blood loss in adult patients undergoing non-cardiac surgeries, the number of transfused red blood cell (RBC) units remains high in developed countries. In addition, the number of possible blood donors between 18 and 65 is consistently decreasing [1]. This may imply increasing costs for the preparation of blood units and reduced availability of blood products for other patients because the cross-matched blood units are assigned only to one patient at a given time. Furthermore, in oncological patients, it is even more challenging to predict the amount of transfused blood and to reduce unnecessary preoperative blood orders because of the different tumor conditions under which the patients receive blood products, such as the diverse grading and stages of tumors, including invasion in surrounding tissue, and presence of metastasis [2]. Extra-abdominal soft tissue sarcoma (STS) further complicates the situation because of their different localization on the body, relationship to the important vessel and nerve structures, and previous treatment with radiation therapy [3].

Therefore, this study aimed to evaluate the possible costs associated with non-transfused blood units for high-grade extra-abdominal STS surgeries. Furthermore, this study aimed to reveal the potential prognostic factors for blood transfusion and use the results to develop a risk prediction score model that can identify a group of patients in which the cross-matching can be safely evaded, thus creating an opportunity for considerable cost savings. The created score model is named “TRANSAR” as an acronym for “Tran” (Transfusion) and “Sar” (Sarcoma).

## Patients and methods

### Patients

This was a retrospective review of 309 consecutive patients with extra-abdominal STS, surgically resected between August 1, 2012, and December 31, 2014, at the Sarcoma Center of University Hospital of Essen (excluded incisional biopsies). The ethics committee approved this study at our institution. All data were anonymized entirely and complied with all local data protection requirements.

### Histology and tumor size

Before definitive surgical resections, all tumors were biopsied, and a pathologist performed the histopathological classification according to the World Health Organization classification of tumors of soft tissues [4]. The indications to perform surgical resection were provided by interdisciplinary tumor boards, which consisted of the medical specialists of departments of surgery, medical oncology, radiation oncology, pathology, and radiology, according to the histology of the tumors and radiological scans of magnetic resonance imaging and computed tomography. All tumor resection and reconstruction surgeries were performed by three surgeons with surgical expertise in levels IV and V in the treatment of STS [5]. Absolute tumor sizes were measured by pathologists postoperatively during the histopathological examination.

### Transfusions

Anesthesiologists determined the indication for preoperative cross-matching according to assessing the patient’s physiological reserves and risk factors. Transfusions were performed according to the German guidelines for blood transfusion, intraoperatively by anesthesiologists and postoperatively by surgeons [6]. There were no preoperative blood transfusions. Pretransfusion compatibility was performed automatically using the “Erytra automated system for blood typing.” Crossmatch/transfusion (C/T) ratio was defined as the ratio of cross-matched packed blood units for potential transfusion to the number of units transfused.

### Costs of pretransfusion processes

Pretransfusion processes, including blood testing and informed consent, were included to evaluate economic loss because of non-transfused patients. According to previous studies, the costs associated with pretransfusion processes varied between 15 and 25% of the total cost of a blood transfusion in different hospitals [7]. The population-weighted mean cost of transfusion of two blood units in Western Europe was estimated at 877.69 €, or 438.8 € per unit, according to previous studies [8].

### Statistical analysis

#### Preoperative prediction

A binary classification method was used to evaluate the preoperative prediction of blood transfusion. Sensitivity (true-positive rate) was defined as the proportion of blood transfusions (true-positives) that were preoperatively correctly identified and or which cross-matching was performed. Specificity (true-negative rate) was defined as the proportion of the cases without transfusion (true-negatives) that did not need preoperative cross-matching. Receiver operator characteristics (ROC) curves were not generated because a single binary predictor variable was used.

#### Design of scoring system

A statistical algorithm for increasing the number of minority cases in a balanced data was performed to limit imbalance bias in our model. Afterward, the following variables were included in the multivariate logistic regression analysis to define independent predictors for blood transfusion: age, hemoglobin count, sex, tumor location and size, histology, neoadjuvant radiation therapy, or radiation therapy for the previous tumor in cases of tumor recurrences, resection margins, therapy with isolated limb perfusion with tumor necrosis alpha and melphalan, number of recurrences, ASA-score (American Society of Anesthesiologists), presence of coronary heart disease. Linearity was tested and assessed using the Box-Tidwell procedure [9]. Bonferroni-correction was applied to all variables in the model. The data set was assigned a randomized 80/20 split into training and testing cohorts for scoring development. Prior to building a binary classification scorecard model, variable screening and exploratory data analysis with the help of weight of evidence (WOE) and information value (IV) were performed [10, 11]. WOE and IV are simple but powerful and widely used techniques to perform variable transformation and selection in scoring to measure the separation of good and bad variables.

The values of the IV statistic for variable selection can be used as follows: less than 0.02: the predictor is not helpful for modeling; from 0.02 to 0.1: the predictor has only a weak relationship to the good/bad odds ratio; from 0.1 to 0.3: the predictor has a medium-strength relationship to the good/bad odds ratio; more than 0.3: the predictor has a strong relationship to the good/bad odds ratio. Variables with low predictive power as measured by IV were removed (IV < 0.02). For continuous variables (hemoglobin count), a coarse classing after fine classing was estimated. Scorecard scores for variables were calculated based on the results from WOE. All scores of WOE were summarized to a total score that can be used for risk stratification. The score was used in a logistic regression model to estimate the coefficient of the score. Then, the regression equation was used to predict the probability of outcome events, given the score of individual patients. The relationship between scores and the probability of outcome events was visualized in a barplot. The outcome of the classification model was described as a receiver operator characteristics (ROC) curve. The area under the ROC curve was used to measure the classification model’s quality. Statistical analysis was performed with SPSS (Statistical Package for the Social Sciences) software, version 23.0, and R Statistical Software (version 2.14.0; R Foundation for Statistical Computing, Vienna, Austria) using a step-by-step tutorial for the development scoring system for risk stratification in clinical medicine [12].

## Results

### Patients’ characteristics

Demographic and clinical characteristics of the patients with extra-abdominal STS are shown in Table 1.

**Table 1:** Demographic and clinical characteristics of the patients with extra-abdominal STS

Variables		Patient
Age		57 (18–89)
Sex	Male	152 (49.2%)
	Female	157 (50.8%)
Localization		
	Head/neck	5 (1.6%)
	Thoracic trunk	49 (15.9%)
	Abdominal trunk	17 (5.5%)
	Axilla	9 (2.9%)
	Upper arm	20 (6.5%)
	Forearm	18 (5.8%)
	Hand	5 (1.6%)
	Gluteal	14 (4.5%)
	Upper leg	97 (31.4%)
	Lower leg	31 (10%)
	Foot	14 (4.5%)
	Pelvis	9 (2.9%)
	Groin	21 (6.8%)
Tumor size		
	≤ 5 cm	98 (31.7%)
	> 5 cm to ≤ 10 cm	140 (45.3%)
	> 10 cm to ≤ 15 cm	42 (13.6%)
	> 15 cm	28 (9.1%)
Depth		
	Superficial	23 (7.4%)
	Deep	286 (92.6%)
Grading		
	Low grade	59 (19.1%)
	High grade	250 (80.9%)
Hemoglobin count		13.5 (6.5–18.40)
Presence of coronary heart disease		
	Yes	28 (9.1%)
	No	281 (90.9%)
309 Patients		

Out of 309 patients with extra-abdominal STS, the preoperative cross-matching was performed for 206 patients, leading to ordering 838 blood units. For 70 patients, two units per patient were reserved. Similarly, for 75 patients, four units; for 49 patients, six units; for eight patients, eight units; and for four patients, ten units of blood were prepared. However, a total of only 92 units of blood were transfused in 31 patients, either intraoperatively or postoperatively (< 24 h), of which five patients received one unit each, 16 patients received two units each, four patients received three units each, three patients received four units each, and one patient received eight, 11, and 12 units each. The sensitivity and specificity of preoperative prediction of blood transfusion were 0.22 and 0.37 retrospectively (Table 2).

**Table 2:** Preoperative prediction of blood transfusion with a sensitivity, specificity, precision, accuracy, and F1 score of 0.22, 0.37, 0.15, 0.43, and 0.18 retrospectively

	Preoperative prognoses and cross-matching	
Transfusion	True-positive	False-negative
	Ja /Ja	Ja/Nein
	31	0
	False positive	True negative
	Nein/Ja	Nein/Nein
	175	103
Sensitivity = true positive/ (true positive + false negative) Sensitivity = 31/(31 + 103) = 31/144 = 0.22		
Specificity = true negative/(true negative + false positive) Specificity = 103/103 + 175 = 0.37		
Precision = true positive / (true positive + false positive) Precision = 31/(31 + 175) = 0.15		
Accuracy = (true positive + true negative)/total number of prediction Accuracy = 31 + 103/309 = 0.43		
F1 Score = 0.18		

### Costs of pretransfusion processes

The difference between cross/matched and transfused blood was 746 units, with a C/T ratio of 9.1.

The economic loss in this case was:

- 746 non-transfused blood units × (438.80 € × 15)/100 = 49,101.72 €, if pretransfusion processes comprised of 15% of the total costs of blood transfusion,
- or,
- 746 non-transfused blood units × (438.80 € × 25)/100 = 81,836.20 €, if pretransfusion processes comprised of 25 % of the total costs of blood transfusion.

### Model development and performance

**Fig. 1:**
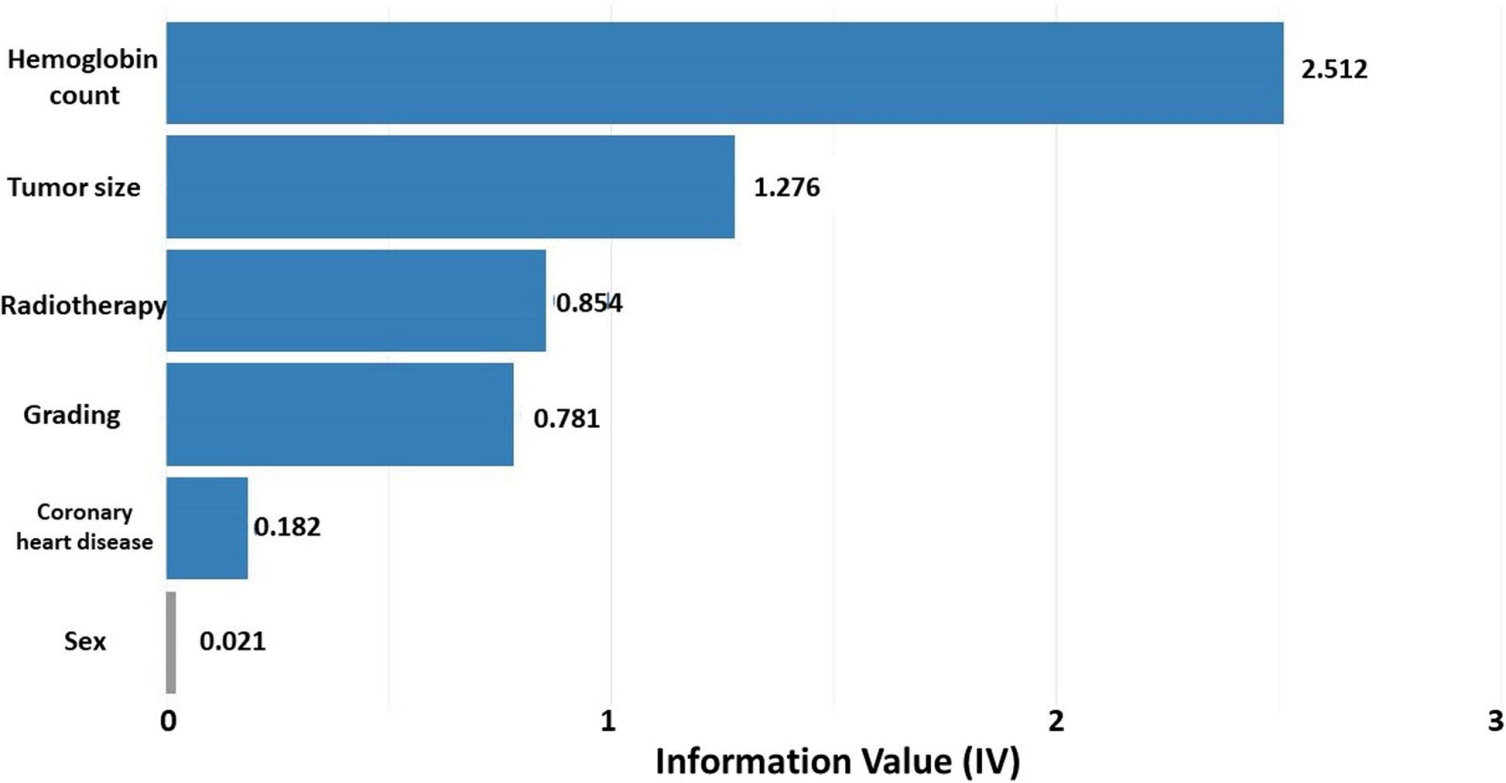
Information value for variables. Variables with high predictive power (IV > 0.02) were selected for the development set

**Fig. 2:**
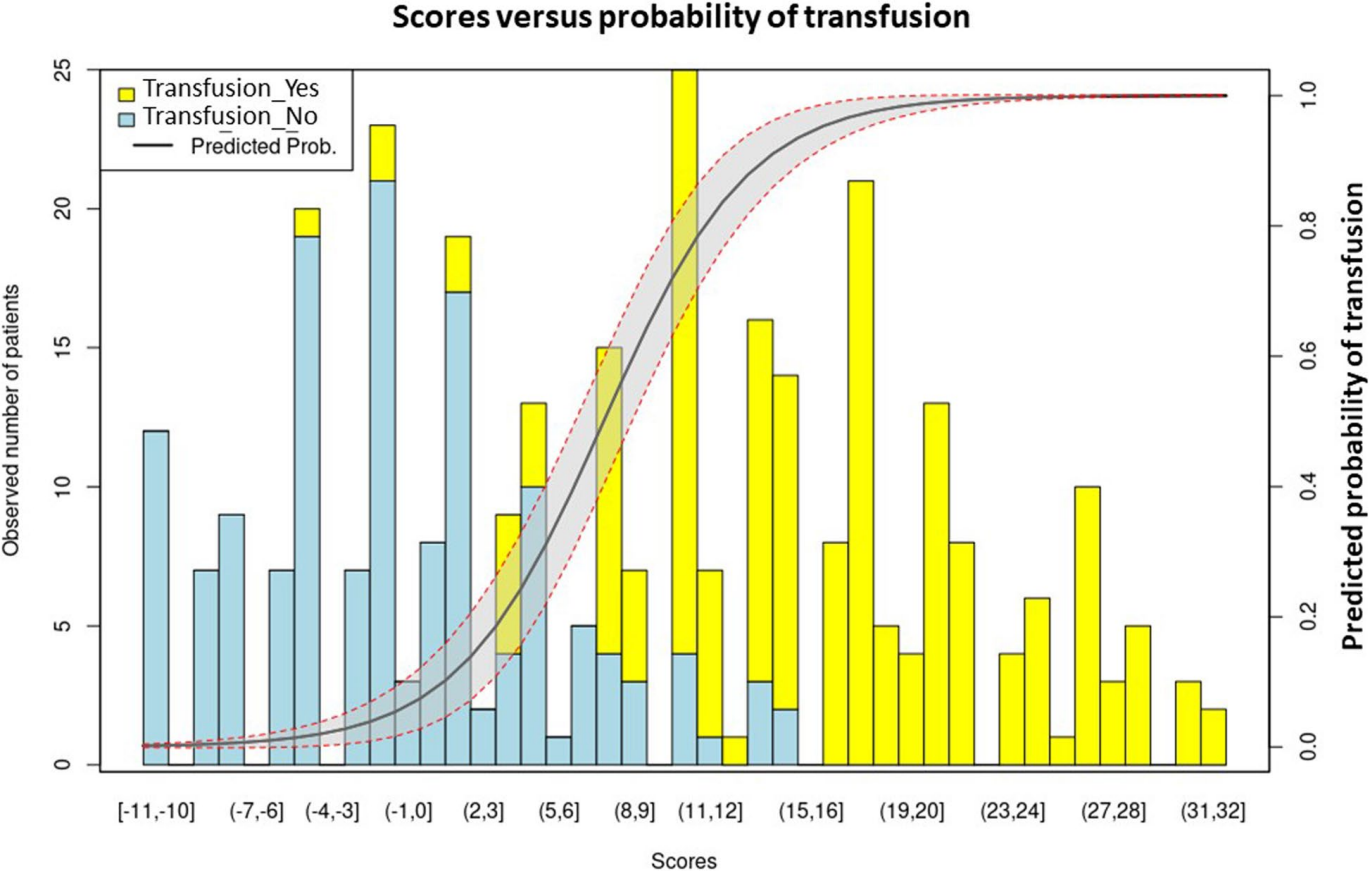
The relationship between transfusion risk score and probability of transfusion in the development set

**Fig. 3:**
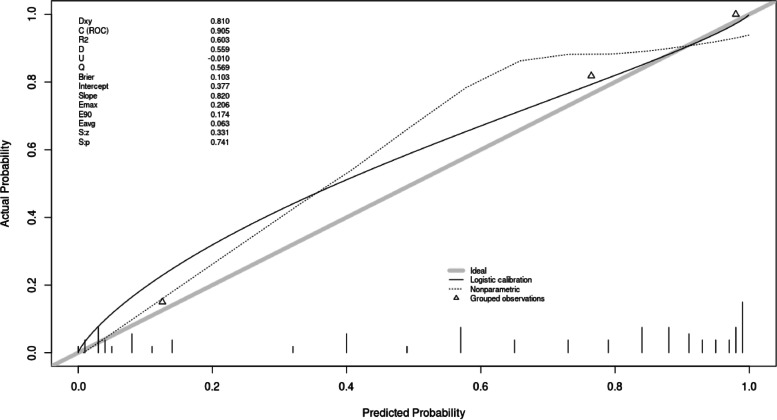
The predicted probability is plotted against the observed probability. The ROC analysis of TRANSAR showed the calculated area under the curve (AUC) of the five evaluated variables by 0.905. Dxy—Somer’s Dxy rank correlation between *p* and *y* [2(C − .5), C = ROC area]; *R*^2^—Nagelkerke-Cox-Snell-Maddala-Magee *R*-squared index; D—Discrimination index D [ (logistic model L.R. *χ*^2^ − 1)/*n*], L.R. *χ*^2^, its *P*-value; U—Unreliability index, *χ*^2^ with 2 d.f. for testing unreliability (H0: intercept = 0, slope = 1), its *P*-value; Q—the quality index; Brier score—average squared difference in *p* and *y*; intercept and slope; Emax—maximum absolute difference in predicted and loess-calibrated probabilities; Eavg—the average in same; E90—the 0.9 quantile of same; Sz/Sp—Spiegelhalter *Z*-test for calibration accuracy, and its two-tailed *P*-value

All variables were found to follow a linear relationship. Correlations between predictor variables were low (*r* < 0.70), indicating that multicollinearity was not a confounding factor in the analysis. The binomial logistic regression model was statistically significant, *χ*^2^(5) = 350.514, *p* < 0.001, resulting in a large amount of explained variance, as shown by Nagelkerke’s *R*^2^ = 0.799 [13]. Out of the variables entered into the regression model, five were selected with a high predictive power using the concept of IV: hemoglobin count (information value—2.512), tumor size (information value—1.276), radiation therapy (information value—0.854), presence of coronary heart disease (information value—0.781), grading (information value—0.182) (Fig. 1). After fine classing hemoglobin count was coarse classing into five subgroups: less than 10.4, from 10.4 to 12.1, from 12.2 to 13.5, from 13.6 to 14.8, and more than 14.8 (Supplementary File). After that, a scorecard model for variables was developed based on the results from WOE (Table 3). The relationship between scores and the probability of outcome events was visualized in a barplot, and there was an increased risk of blood transfusion with a higher score (Fig. 2). The ROC analysis of TRANSAR showed the calculated area under the curve (AUC) of the five evaluated variables by 0.905 (Fig. 3).

**Table 3:** Multivariate statistical analysis of significant predictors for transfusion and corresponding weights for TRANSAR score

Predictor	Group	WOE	Score weight
Tumor size	≤ 5 cm	− 2.352	− 7
	> 5 cm to ≤ 10 cm	− 0.368	− 1
	> 10 cm to ≤ 15 cm	1.172	3
	> 15 cm	1.742	5
Grading	High-grade	0.141	0
	Low-grade	− 0.917	− 3
Radiotherapy	No	0.050	0
	Neo-adjuvant	1.297	3
Hemoglobin	0 ≤ 10.4	2.779	8
	10.4–12.2	0.518	1
	12.2–13.6	− 0.656	− 2
	13.6–14.8	− 1.909	− 5
	≥ 14.8	− 2.988	− 8
Presence of coronary heart disease	Ja	2.028	8
	Nein	− 0469	− 2

The created score was named “TRANSAR” as acronym for “Tran” (Transfusion) and “Sar” (Sarcoma)

## Discussion

Patient blood management is an evidence-based perioperative multidisciplinary and multimodal patients-specific team approach, which consists of three pillars that optimize the volume of blood transfusions [14, 15]. The preoperative and intraoperative steps of the second and third pillars include the identification of bleeding risks, assessment and optimization of the patient’s physiological reserve and risk factors, and meticulous hemostasis and surgical techniques. Because extra-abdominal high-grade STS are rare heterogeneous tumors with variable presentations, localizations, behaviors, and outcomes [16], it is challenging to implement the steps mentioned above of evidence-based knowledge. This was also demonstrated in the results of our retrospective analysis, which revealed a very high coefficient of C/T ratio and a high economic loss.

Therefore, we attempted to solve this problem by analyzing our clinical data to reveal the potential prognostic factors for blood transfusion and to create a risk prediction score model that can predict blood transfusion to patients with high-grade extra-abdominal STS with high probability. The TRANSAR score can be deployed anywhere and managed effortlessly.

The results of our study indicate that in most cases, cross-matching would be needed for patients with coronary heart disease, which could be associated with perioperative antiplatelet management strategy, including continuation until surgery, or was not stopped during surgery. In addition, this could also be associated with an increased volume of transfusion and blood loss. Moreover, an increased risk of re-operation because of postoperative bleeding, as well as an increased length of hospital stay [17–19]. In this study, the preoperative anemia in patients with STS varied between 20 and 30%. The cause of tumor-related anemia may depend on the dysfunction of iron metabolism, inadequate production of erythropoietin, reduced number of erythroid progenitor cells in the bone marrow, and the production of inflammatory cytokines [20, 21]. Additionally, low preoperative hemoglobin concentration could be explained by the tumor’s paraneoplastic effects or the influence of neoadjuvant chemotherapy as a part of multimodal oncological treatment [22, 23].

The tumor size and grading could be associated with higher blood loss during tumor resection because of possible hypervascularity, surgical challenges, and extended operating time [24, 25]. Our study demonstrates that neo-adjuvant radiation therapy or radiation therapy for a previous tumor can increase the risk of excessive blood transfusions and thus can be associated with fibrosis or soft tissue edema due to radiation toxicity, further complicating the surgical procedure [26, 27].

The findings of this study have to be interpreted in light of some limitations. The first limitation is the small size of the patient group and retrospective study design because of the rarity of these malignancies in the population. Because of these reasons, it was challenging to create a score that could predict the number of necessary blood units to be cross-matched. A multicenter prospective study utilizing this training model’s data and even the possibility of real-time constant model retraining could solve this problem. The second limitation concerns the differences in the expertise of operating surgeons, which can affect the amount of blood loss. Therefore, this study’s results and applications could apply only to high-volume sarcoma centers with surgical expertise in levels IV and V.

Thus, the TRANSAR score is the first attempt to design a prediction model for a blood transfusion by patients with extra-abdominal high-grade STS using a combination of demographic and clinical variables on admission. After summarizing all scores of five prognostic factors, the patients with a score of more than six points have a risk of being transfused more than 50%. However, the clinician makes the final decision to perform a cross-match.

TRANSAR score is a part of evidence-based medicine, which could provide tremendous and advantageous information for clinical practice in treating rare diseases. As the colleagues, Kohane IS et al. published in the New England Journal of Medicine, “biomedical research, data technologies, and clinical care all require resources, but the era of shifting more and more economic resources toward healthcare is going to end.” Therefore, in the future, there could be an increased focus on more efficient use of resources to deliver the best care to the patient at the lowest cost [28].

## Conclusion

We proposed that the use of TRANSAR score for the patients with extra-abdominal STS may reduce the amount of unnecessary cross-matching and thus save costs in health care. The transfusion risk score passed our internal validation successfully. Further external validation as a prospective study in other sarcoma groups is needed. However, this tool is not intended to replace the competence of medical specialists and is aimed only at facilitating clinical decision-making.

## Supplementary Information

**Additional file 1.** Fine classing of the continuous variable (hemoglobin count).

## Data Availability

The data that support the findings of this study are available on request from the corresponding author.

## Abbreviations

C/T-ratio: Crossmatch/transfusion ratio (C/T)
STS: Soft tissue sarcoma
IV: Information value
WOE: Weight of evidence
AUC: Area under the curve
ROC: Receiver operator characteristics
RBC: Red blood cell

## References

[1] Seifried E, Klueter H, Weidmann C (2011). How much blood is needed?. Vox Sang.

[2] Fischer D, Neb H, Choorapoikayil S (2019). Red blood cell transfusion and its alternatives in oncologic surgery-a critical evaluation. Crit Rev Oncol Hematol.

[3] Massarweh NN, Dickson PV, Anaya DA (2015). Soft tissue sarcomas: staging principles and prognostic nomograms. J Surg Oncol.

[4] Jo VY, Fletcher CD (2014). WHO classification of soft tissue tumours: an update based on the 2013 (4th) edition. Pathol.

[5] Tang JB (2009). Re: Levels of experience of surgeons in clinical studies. J Hand Surg Eur.

[6] Biscoping J (2009). Therapy with blood components and plasma derivatives: the current cross-sectional guidelines. Anaesthesist.

[7] Shander A, Hofmann A, Ozawa S (2010). Activity-based costs of blood transfusions in surgical patients at four hospitals. Transfusion.

[8] Abraham I, Sun D (2012). The cost of blood transfusion in Western Europe as estimated from six studies. Transfusion.

[9] Box GEP, Tidwell PW (1962). Transformation of the independent variables. Technometrics.

[10] Baesens B, Rösch D, Scheule H (2016). Credit risk analytics: measurement techniques, applications, and examples in SAS.

[11] Siddiqi N (2017). Intelligent credit scoring: building and implementing better credit risk scorecards.

[12] Zhang Z, Zhang H, Khanal MK (2017). Development of scoring system for risk stratification in clinical medicine: a step-by-step tutorial. Ann Transl Med.

[13] Backhaus K, Erichson B, Plinke W, Weiber R. Multivariate Analysemethoden. Eine anwendungsorientierte Einführung. Springer-Verlag Berlin Heidelberg. 2016. 10.1007/978-3-662-46076-4.

[14] Isbister JP (2013). The three-pillar matrix of patient blood management–an overview. Best Pract Res Clin Anaesthesiol.

[15] Leahy MF, Hofmann A, Towler S (2017). Improved outcomes and reduced costs associated with a health-system-wide patient blood management program: a retrospective observational study in four major adult tertiary-care hospitals. Transfusion.

[16] Brennan MF, Antonescu CR, Moraco N, Singer S (2014). Lessons learned from the study of 10,000 patients with soft tissue sarcoma. Ann Surg.

[17] Berger JS, Frye CB, Harshaw Q (2008). Impact of clopidogrel in patients with acute coronary syndromes requiring coronary artery bypass surgery: a multicenter analysis. J Am Coll Cardiol.

[18] Chu MW, Wilson SR, Novick RJ (2004). Does clopidogrel increase blood loss following coronary artery bypass surgery?. Ann Thorac Surg.

[19] Burger W, Chemnitius JM, Kneissl GD, Rücker G (2005). Low-dose aspirin for secondary cardiovascular prevention - cardiovascular risks after its perioperative withdrawal versus bleeding risks with its continuation - review and meta-analysis. J Intern Med.

[20] Szkandera J, Gerger A, Liegl-Atzwanger B (2014). Pre-treatment anemia is a poor prognostic factor in soft tissue sarcoma patients. PLoS ONE.

[21] Grimer R, Gaston C, Carter S (2013). The relationship between pretreatment anaemia and survival in patients with adult soft tissue sarcoma. J Orthop Sci.

[22] Van Belle SJ, Cocquyt V (2003). Impact of haemoglobin levels on the outcome of cancers treated with chemotherapy. Crit Rev Oncol Hematol.

[23] Knight K, Wade S, Balducci L (2004). Prevalence and outcomes of anemia in cancer: a systematic review of the literature. Am J Med.

[24] Kawai A, Kadota H, Yamaguchi U (2005). Blood loss and transfusion associated with musculoskeletal tumor surgery. J Surg Oncol.

[25] Thompson PA, May D, Choong PF (2014). Predicting blood loss and transfusion requirement in patients undergoing surgery for musculoskeletal tumors. Transfusion.

[26] Davis AM, O'Sullivan B, Turcotte R (2005). Late radiation morbidity following randomization to preoperative versus postoperative radiotherapy in extremity soft tissue sarcoma. Radiother Oncol.

[27] O'Sullivan B, Davis AM, Turcotte R (2002). Preoperative versus postoperative radiotherapy in soft-tissue sarcoma of the limbs: a randomised trial. Lancet.

[28] Kohane IS, Drazen JM, Campion EW (2012). A glimpse of the next 100 years in medicine. N Engl J Med.

