# Supplementary figures and images for: Optimization of the preoperative requirements of blood units for the surgical treatment of extra-abdominal soft tissue sarcoma: the TRANSAR score
Source: World J Surg Oncol. 2022 Dec 4;20:378. doi: 10.1186/s12957-022-02839-0 (PMC9721031; doi:10.1186/s12957-022-02839-0)

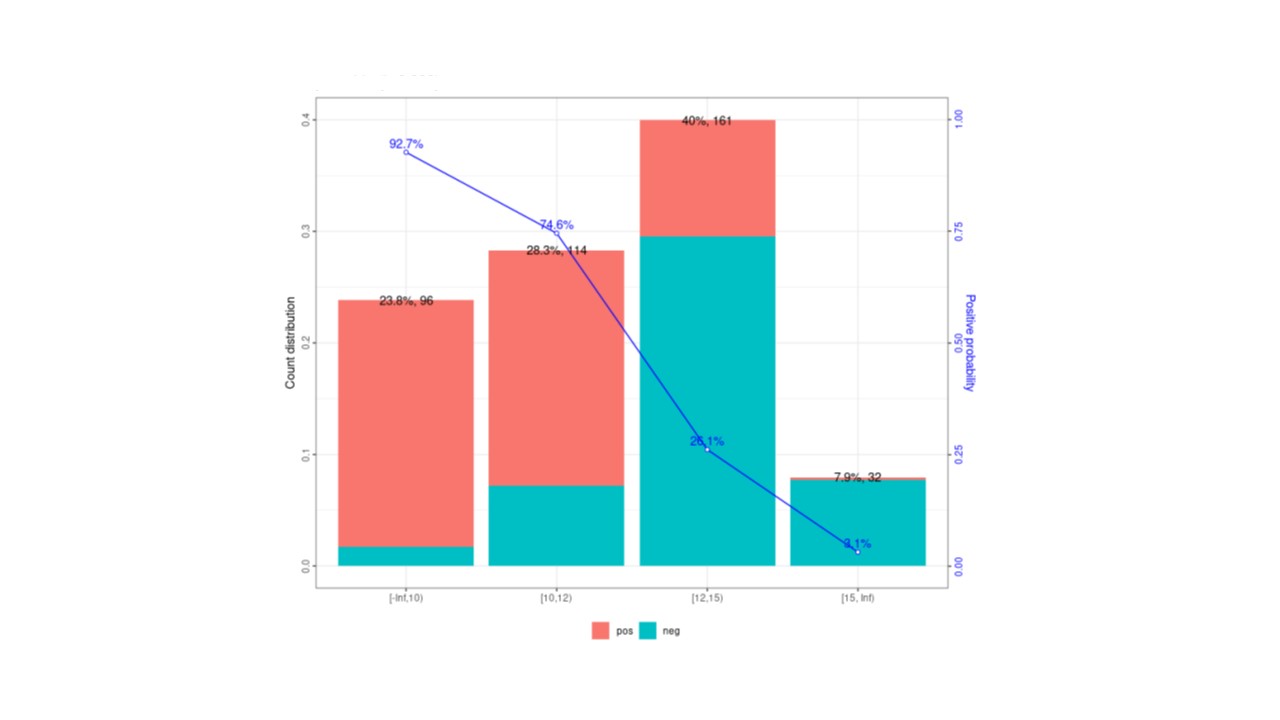

Supplement: Supplementary file 1 — Additional file 1. Fine classing of the continuous variable (hemoglobin count). [file 12957_2022_2839_MOESM1_ESM.jpg]
